# Some Developments in Dermatology during the Last Thirty Years

**Published:** 1928

**Authors:** W. Kenneth Wills

**Affiliations:** Physician in Charge of the Skin Department, Bristol General Hospital


### The Bristol Medico-Chirurgical Journal

"*Scire est nescire, nisi id me
Scire alius sciret.*"

SPRING, 1928.

### SOME DEVELOPMENTS IN DERMATOLOGY DURING THE LAST THIRTY YEARS.

Presidential Address, delivered on 12th October, 1927, at the opening of the fifty-fifth Session of the Bristol Medico-Chirurgical Society.

BY

W. Kenneth Wills, O.B.E., M.B., B.Ch.,

*Physician in Charge of the Skin Department, Bristol General Hospital.*

It is my first duty and privilege to thank you for the honour you have conferred upon me by electing me to this Chair. This is the greatest honour that you have in your power to confer upon any of your members, and it is a matter of great pride to me that you have considered me worthy to occupy the Chair which has been so ably filled by my predecessors.

During the last thirty years none of your Presidents has addressed this Society on a purely dermatological subject.

Dr. A. J. Harrison in 1894 dealt with "Comparative Pathology," as illustrated by experience of the animals in the Clifton Zoological Gardens.

Dr. Henry Waldo in 1907 addressed the Society on

"Syphilis," just two years after the discovery by Schaudinn and Hoffmann of the Spirochæta pallida. He took the opportunity of noting the advance made by pathologists, and urged a closer connection between the clinic and the laboratory.

In 1923 Dr. Nixon directed our attention to the debt that medicine owed to the fine arts.

I have therefore thought that it may prove of some service to pass in review some developments in dermatology during the last thirty years.

This period is marked by a considerable development of the pathological investigation of skin diseases. It has been said that the successful dermatologist is he who understands the "skin and everything inside it." During these years much more attention has been concentrated upon functions of the body other than of the skin itself, in the attempt to understand the phenomena of skin complaints. Not only has the microscope been at work on the skin, but the batteries of the biochemist, the bacteriologist, the vaccinist, have been brought to bear upon the patient as a whole, and many cutaneous phenomena are now better understood as symptoms of a general diseased condition than as a local disturbance of the skin.

In 1907 Dr. Waldo could do little more than anticipate the profound effect that the discovery of the Spirochæta pallida would have upon the diagnosis and treatment of syphilis. In that very year Wassermann announced his "non-specific complement fixation test," and this with its subsequent developments has added enormously to our powers of diagnosis; so that, while much has to be found out yet to make this the reliable instrument we require, we have to acknowledge that we should be very poorly equipped in our treatment of syphilis without the guide which it affords.

In 1909 Ehrlich introduced salvarsan, which has led to a vast activity in the laboratories in producing endless preparations for the cure of syphilis. Some of these are proving by results to be curative of the disease in early stages with the minimum of damage to the patient.

In syphilis, therefore, we have advanced during the period under our review, not alone in the methods of diagnosis, wherein we are able to state definitely by dark-ground field that Spirochæta pallida is present, and that a sore is syphilitic, but we can thereby bring the case under treatment early enough for cure to be possible by one of the new remedies which lie to our hand.

By treatment we can also prevent the onset of the secondary manifestations of the disease so effectively that students have now little chance of studying the cutaneous phenomena which formed so large a part of the clinical material in the skin clinics of my student days. It is a matter of great importance to the public health that the infectivity of these early cases can be prevented, or at least arrested, early by these injection treatments, and thus indiscriminate spread of syphilis has decreased materially. The later symptoms characteristic of the so-called tertiary stage may often be healed rapidly and effectively.

It is when we come to consider the lesions of the central nervous system that we have to admit that all our hopes have not been realised. Indeed, there are evidences adduced which are, as yet, little more than impressions and rumours, that lesions of the central nervous system are more prevalent now than they used to be, and it is openly stated that this is due to treatment by salvarsan and its derivatives. On the other hand, statistics have been brought forward to show that there are fewer cases of dementia paralytica, especially in women, and it is suggested that this is due to the shortened period of infectivity due to salvarsan derivatives. Yet while no one would hesitate to advise and carry out treatment which has such immediately beneficial results, it must be borne in mind that the remote effects of syphilis and its treatment may still be far-reaching and defy our best present endeavours.

I need not remind you of the attempts to affect the central nervous system by means of pyrexia-producing measures such as malaria and protein shock, but this carries me too far from my immediate purpose.

The term "protein shock" introduces another modern development, use of which is made in dermatology. Indeed, an entirely new vocabulary has been instituted bristling with terms such as "anaphylaxis," "acidosis," "sensitisation," "hostile protein," etc., all of which have their interest to the dermatologist.

The modern theories of *urticaria* exemplify in a concrete form the advances which have been made in the study of dermatology in recent years. No longer are we content to leave the subject of ætiology to be expressed by vague terms such as drugs and poison. We want to know what poison and the method of its working. The modern tendency is to explain urticaria as a phenomenon of anaphylaxis.

Anaphylaxis may be defined as a state presenting phenomena which arise in a sensitised animal as the result of the absorption of a subsequent dose of the same protein as was the original sensitising agent, or one nearly akin to it. There is evidence that urticarial lesions, both of external as well as internal origin, may be regarded as of anaphylactic nature. It has even been shown by Cranston Low that insect bites may reveal a sensitisation, and the severity of the wheals be dependent upon a previous inoculation by such insects.

Where urticaria is due to an internal cause, we shall probably all admit that foods, such as fish and pork, eggs and certain fruits, which are liable to produce wheals or even constitutional disturbance akin to serum-sickness, can excite anaphylactic symptoms. These symptoms are due to foreign substances introduced from without the body; but there are also well-known instances of urticaria which have a relationship to hydatid cysts, menstrual troubles or operative interference, and even such injuries as may produce hæmatomata have been known to be followed by urticarial attacks. These may be regarded as anaphylaxis induced by proteins absorbed from the patient's own tissues which are acting as foreign substances, to which the patients are sensitised.

Not only so, but microbic organisms which are so placed in the body that their toxins are readily absorbed into the blood-stream may produce urticaria. To quote Cranston Low[1] again: "Focal infections, such as tonsillitis, pyorrhœa, abscesses at the roots of teeth, infections of the bladder and bowel, etc., have all been known to produce urticaria."

The removal of these sources of infection has cured the skin conditions. This may explain why urticaria occurs at times in cases of acute rheumatism, of malaria, of influenza or of pneumonia. The extraordinary phenomena seen in angioneurotic œdema, in which the swelling arises so suddenly and to such an intense degree as to cause danger to life, may be explained by anaphylaxis.

I once saw a patient, years ago, in whom the right side of the face advanced at me while I was speaking to her. One half of her forehead fell in a fold over her eyebrow, the eyelids lay in pouches on the cheek, the cheek fell in a dewlap over the mouth, and the half lips fell sagging below the chin. The tongue was unaffected. In about ten minutes all had disappeared to normal again. This was before "sensitisation" had been suggested as an explanation, and I was quite at a loss to account for it. But she had rheumatism, and as great improvement followed on the administration of sodium salicylate, it may well have been of rheumatic or tonsillar origin.

Although so many cases of urticaria may be attributed to anaphylaxis, there are quite a number which cannot be thus explained. Among these are those cases which are associated with exophthalmic goitre. But even here it has been urged that the Graves's disease is secondary to sepsis, or at least that the normal control of the thyroid excretion upon septic toxins is defective. But there are still to be explained those cases of urticaria which attend the passage of a uterine sound, or seem to be the result of nervous emotion and fright, or that of the man whose hands were affected after the prolonged use of a mechanical vibrator. Such cases would tend to show that the nervous mechanism is affected, and the question as to what cells are affected—whether primarily the capillaries, the nerves, the endocrine glands or the blood with the development of such chemical irritants as histamine—has yet to be answered satisfactorily.

But we may note that we have travelled fast and far from the position of thirty years ago, when the explanation of a poison in the blood had to suffice. Even Wright's calcium deficiency theory no longer satisfies us.

The wall of our ignorance has been set back a little farther, and there are plenty of eager explorers who are doing their best to look over that wall.

When I alluded to urticaria as having been caused by microbic infections, I at once thought of other diseases which had been found to be associated with similar conditions. It has been found that the histological and local bacteriological examinations of skin lesions themselves have yielded little advance in the ætiology of such complaints as lupus erythematosus, psoriasis, dermatitis herpetiformis and pemphigus.

*Lupus Erythematosus.*—Evidence has been adduced that this complaint is dependent upon the action of toxins circulating in the capillaries but elaborated elsewhere. This disease attacks exposed parts, mainly face and hands, producing an erythema which relapses repeatedly and leaves a permanent scar of a most unsightly and distressing nature. It has long been recognised that actinic light is a factor in producing the cutaneous symptoms, but what activated the skin and made it susceptible to the action of light was unknown. Early it was supposed to be a tuberculide, but there are insufficient grounds for connecting all cases of the disease with tuberculosis. As far as this country is concerned, the lessened incidence of tuberculosis has not been attended by any appreciable decrease in the number of erythematosus cases.

We had to be content with the vague term "toxæmia" to indicate a hypothetical toxin in the circulation until Barber, in 1915, brought forward evidence to show that in one case under his control streptococci in the intestine were the source of the toxin. Since this observation has set us looking for foci distant from the skin itself, numerous cases of the disease have been treated successfully by removal of the septic focus, or by vaccines from organisms obtained from tonsils, teeth, antra, sputum, fæces, etc., with immediate relief of the active cutaneous lesions and cessation of recurrences. While it may be too early to state that streptococci are the only organisms capable of producing this predisposing toxæmia, one may note thankfully the great advance which has been made by the discovery of the "focal toxin" factor in a disease which has previously proved so intractable.

*Alopecia Areata.*—The theory of "focal toxin" has been adduced to account for this hitherto unexplained phenomenon. When it is considered how poisonous to hair streptococci are (*cf.* erysipelas, scarlet fever, puerperal fever, pneumonia, and certain influenzal epidemics where possibly streptococci are responsible), it would not be surprising if streptococci in foci like the tonsils, etc., were found to be the exciting cause. Without discussing whether the organisms produce toxins which act upon the nerve endings (thus uniting the toxic, microbic and nerve theories), or whether they act upon the hair roots directly, it should be noted that some cases have been reported in which rapid improvement has attended the use of vaccines from a remote focus.

To quote a case from my own clinic: The patient had a tonsillitis early in December. On Christmas Day the hair fell off in patches. He attended at the hospital in January, and after a few weeks' treatment by streptococcal vaccines obtained from the tonsils by Dr. Hadfield, the hair began to grow freely at the beginning of February. The extraordinary rapidity of the growth suggests that the vaccines were directly responsible. "The time," as Sir Michael Foster used to say, "is not yet ripe for dogmatic statement," but there is certainly an indication that we are moving in the right direction with regard to the effective treatment of this common and troublesome complaint.

*Psoriasis.*—I wish that I could report that a similar advance had been made in the treatment of psoriasis. It is true that the Wassermann test has been useful in eliminating the syphilitic cases where there has been doubt, that X-rays and actinic light have proved of temporary benefit in removing individual patches, but we are still unable to prevent the onset or sometimes to control the disease. One observation has been made which may lead to future developments. Accidental acute suppuration has been followed by the clearing up of the lesions. In a case of my own the patient was admitted to hospital with a general exfoliation consequent on extensive psoriasis which perhaps had been too vigorously treated. After some improvement had occurred from simple measures, exposures to the Tungsten arc lamp were begun. After further improvement he was given quinine, when his hands and forearms suppurated violently, his temperature rose, and his general appearance was one of toxæmia. In about three days the symptoms cleared up, and with this the psoriasis completely disappeared. Unfortunately, fresh small lesions appeared later, after his discharge from hospital, but this is a striking instance that something connected with the process of suppuration is inimical to the psoriatic process. Attempts have been made to influence the complaint by the injections of foreign proteins, and even by injections of the patient's whole blood, but without much success.

An interesting observation has been made by several observers, and recently by Civatte, that columns of leucocytes rise from the tops of the papillæ and spread out under the keratin layer, and it is supposed that these may have some hand in producing the parakeratosis which is the main feature of the complaint. No organisms have been found in the skin or blood which will account for this phenomenon, and until a satisfactory reason can be found for this migration of leucocytes, the cause of psoriasis will presumably remain unknown.

*Eczema.*—Here some advance has been made In the first place several distinct diseases have been completely separated from this heading. For instance, *eczema marginatum* has now appeared under its proper classification of tinea. Cheiropompholyx has since been classified as a distinct clinical entity, and even this again has been shown to be due to two or more causes, one of which is a tineal infection.

Again, the large group of seborrhœic eczema has been separated from the main heading, until now there remains very little of what may be termed, for want of better knowledge, "eczema."

It may be well to remind ourselves that it has been sought to explain that catarrhal condition of skin termed "eczema" by three theories: (1) the diathetic —whereby the toxins associated with the gouty or arthritic state were held responsible for the skin condition (French school); (2) the nervous theory— to account for those cases which seemed to develop from overwork, shock and overstrain; (3) the bacterial infection theory—which was advanced by Unna, who described a coccus as the infective cause. This is now regarded as a harmless denizen of all skins. Since then further theories have been advanced, including (4) the faulty digestion or assimilation theory, whereby the split products of proteins or more possibly fats (fatty acids) are considered as playing a part in the origin of skin catarrh, (5) and the skin sensitisation theory, whereby it is sought to explain the acute exacerbations of eczema and their rapid retrogressions.

I have myself tried to show how these eczemas develop in persons as a direct "infection" from dry dandruff in some individual who is in contact with the patient, if not from the patient himself. From clinical observation I have noted that this is almost universally applicable to all eczemas in children, and to a large majority of adult cases as well.

Dandruff itself comes and goes in a peculiar manner —at one time being so noticeable as to send the patient for treatment, while perhaps in a few days all trace of the dandruff may be gone. But the tendency to grow dandruff is found in families, and is by such regarded frequently as a normal condition of the scalp, whereas there are other families in which it is unknown. The marriage of a dandruff infected person with a clear-scalped individual may lead (and often does) to the infection of the latter. The children of this union will show eczema, especially if the mother is the infected one. If the father has it, and not the mother, the child will or will not show eczema according as to whether he has much or little to do with the child. Thus one family has been observed by Dr. Gee where the father was the source of the infection. When the first child was born the man frequently nursed the infant, who got eczema as the result. With the second and third children the novelty had worn off, and he had little to do with them, and they escaped. But with the fourth child the man was out of work, and was turned on to do nurse, and accordingly this child got eczema. I have observed that the parts affected in children are the exposed parts—face and neck, hands, forearms and popliteals. In school children the backs of the necks and ears may be affected, as though the school teacher had dropped scales on leaning over them. I have known even a small infant of only fourteen days old which developed a patch on that cheek which was uppermost on the nurse's arm. The nurse it was who had the dandruff in this case, and not the parents. Since the fashion of wearing the hair short has come in, the parts affected by the eczema are higher up than they used to be, viz. the upper chest and shoulders, instead of interscapular and sternal regions.

Any cause of itching may lead to eczema development when there is a source of infection in the scalp. Thus one is not surprised to find that eczema developed from a flea-bite on the forearm in a clerk who had dandruff, his left arm being free at his work to scratch the head and the right arm, and thus produce the infection, or that a man working in paraffin developed an irritation on his forearms, and on scratching with dandruff-infected nails eczema ensued, and subsequently elsewhere on his body. After operations, burns and other traumata eczema may develop, and this may resist treatment until the scalp is treated too. In fact, this is constantly being observed clinically, but when one is asked to explain why dandruff may exist for years without any known cases of eczema following this is not so easy. It may be that cocci, or whatever the organisms may be, have their toxicity raised by the excreted waste products which pass through the skin glands from time to time, but that periods of non-toxic saprophytic growth may occur. If this waste material is of protein or split product origin (*e.g.* uric acid, butyric acid, etc.), then the "arthritic" and the "faulty digestion" schools may claim these cases. If sensitisation by foods (eggs, chocolate, fruits, etc.) sets up itching, as often happens, subsequent scratching may lead to infection by dandruff cocci, and so eczema be caused apparently by the foodstuffs. In any case it is quite tenable that organisms, as Unna suggested, are the exciting cause of by far the majority, if not in all cases of eczema. Even where a sudden acute generalised attack of eczema occurs, it may be explained by the sensitisation theory, the sensitising having occurred owing to sudden rise in the toxicity of the organisms. It would be well to bear this combined theory in mind, and to bring it to bear upon such cases of eczema as we may meet with.

Any survey of present-day dermatology would be inadequate without a reference to actinotherapy.

During these thirty years we have seen the rise of the Röntgen rays, and it was early that their use was discovered in the treatment of skin disease. The first observation of the action of these rays upon the skin was the accidental burns which were produced with too long exposures in taking radiographs. It was then found that there was some selective action upon young and actively dividing cells, and this action was found to destroy malignant cells while leaving the normal cells unharmed. Unfortunately, the press started an unwarranted boom of the X-rays as the cure of cancer. This of course was not claimed by the medical profession, for it was realised that as the action of light varies inversely with the square of the distance, it is impossible to bring under the effective action of the rays parts which are remote from the tube without giving an overdose to parts which are nearer.

Even with better methods of measurement of dosage, with screens and "cross-fire" and the greatly improved tubes, I doubt if anyone will be found who holds too optimistic a view as to the hopes of the indiscriminate cure of cancer by this method. But locally on the surface the X-rays have added considerably to our powers of healing or controlling various dermatoses.

It should be remembered that all actinic rays have the power of "ionizing" the cells. That is, they liberate electrons from the atoms. After the discharge of an electron the atom becomes electrically charged, and according to Sir Oliver Lodge, "it is no longer neutral or inert, it is actively and chemically fierce: it is no longer satisfied, it seeks to combine with another." This serves to explain any bactericidal power and the chemical and physiological activities of light.

At first X-rays were utilised for the treatment of cutaneous carcinomata, lupus vulgaris and erythematosus, nævi and tinea.

Acting upon the experimental work done on the development of ova, where it was found that a minimal exposure to rays increased mitosis and growth of cells generally, and that beyond this minimal dose growth was inhibited or even arrested, it was sought to bring into action an optimum dose of rays which would destroy the cells of more embryonic type, while leaving the normal tissue cells unharmed.

In early cases of skin carcinomata this has been achieved, but as in so many cases there are always disadvantages as well as advantages.

Where extension has taken place into cartilage or bone the rays seem to be powerless. Too frequent doses of rays may induce a condition which is termed X-ray hardening, where no further reaction of the skin cells can be expected. Also each case has to be watched most carefully for recurrence, as in other methods of treatment. With regard to this, I am not at all sure that some of these so-called recurrences are due always to insufficient treatment. There is some reason for supposing that the tendency to malignant changes which originated the tumour is not lost entirely in the skin cells when the original ulcer has completely scarred. However, even with these defects, X-rays and radium, rightly used, may be regarded as the treatment for rodent ulcers and early skin carcinomata which offers the best prospect of relief with the best cosmetic effect.

With regard to lupus vulgaris, however, it must be frankly admitted that the field of usefulness of the X-rays has contracted considerably. X-rays *will* cure lupus, but the after experience of cases which have had long and frequent exposures shows a disastrous increase in the numbers of carcinomata developing upon the scars. Carcinomata are of course met with in untreated lupus of long standing, but the numbers have increased so much since the rays have been in use, that this treatment in most hands is now used only for the reduction of exuberant masses and the healing of ulcers, in which the results are brilliant. The deep-seated nodules can be treated by other methods which are more rapid, such as by the new selective caustic pyotropin.[2]

The length and number of exposures necessary for the destruction of nævi have made it unsafe to use X-rays in the treatment of these.

With regard to the treatment of tinea tonsurans, however, it may be claimed that the experience of the past twenty years has shown that we have in X-rays a rapid cure of this distressing and persistent complaint. Since this method has been used for school children by the Education Authority the incidence of the disease has been reduced to almost negligible proportions. There are possible accidents to be met with in this treatment. Cases of permanent alopecia were more common earlier on than they are now. Some of these may possibly have been due to some peculiar idiosyncrasy, others to too active antiseptic treatment used in addition to the raying, while others may have been due to faulty technique ; but even with some of these alopecia cases the hair has been restored after a period, even after as long as five years.

It is essential that the treatment be undertaken by a medical man of experience, and that the dosage be timed with great accuracy. It is also wise to have one machine specially tuned for this work and kept exclusively for ringworm cases. With these conditions it may well be that the "small spored" ringworm, which has proved such an economic disaster to schools, may soon be a rarity or perhaps completely stamped out.

Not only have X-rays proved of service in rodent ulcers, tuberculides and tinea tonsurans, but they are of great service in itching, scarring and suppurative diseases. Taking advantage of the fact that after a sufficient dose of rays the nerve-endings are anæsthetised for a period, one has been able to give a temporary relief to cases of anal and vulvar pruritus, to areas of lichenification, to chronic eczema, and lichen planus, and at times this temporary relief has been followed by a cure of the condition.

X-rays have a peculiar effect upon scar tissue, in rendering it more elastic and atrophic. So that where bands of scar are interfering with the free movement of limbs and natural orifices these can be rendered more supple. For instance, I have had a small child under my care who had her mouth so reduced in size by a scald, that movement of the jaws was prevented, and food had to be pushed into her mouth by means of a pencil. After raying the mouth became quite flexible and expansible. Expression returned and mastication became possible once more.

In hypertrophic scars and even fibromata, provided the cases are treated early enough, atrophy can be induced and deformity corrected. But the most dramatic results are seen with true keloid. Here the whole condition can be arrested and the masses reduced to flat, white or slightly pigmented scars. Even in sclerodermia some atrophy may be induced.

In suppuration it has been observed that limitation of the area, more rapid pointing of the abscess, and relief of pain may be the after-effect of X-raying. This can be taken advantage of in boils and carbuncles, but in acne pustulata the best results may be seen. The rays have the power of arresting and diminishing glandular activity, so that in acne, not only do the pustules discharge rapidly and dry up, but the comedones are shed and the grease-glands dried up, so that a cure may be obtained from this method of treatment alone. When also hypertrophic and keloidal scars are reduced, the best cosmetic effects are seen to result from this treatment.

The rays have also been used for hyperidrosis with good effect.

Although the tale of usefulness of the rays has not been exhausted, enough has been said to show the advance which has been made in dermatology by means of the X-rays. And what is true of X-rays is also true of radium, and to some extent to light.

With the improvement in the lamps, more advantage has been taken of the action of ultra-violet and other rays in the treatment of skin conditions. It has been held that light is bactericidal directly, and indirectly through the action of leucocytosis, that it increases metabolism and the absorption of calcium and phosphorus, that it increases hæmoglobin and promotes the activity of the endocrine glands, especially the thyroid and adrenals, and that it counteracts the effects of anaphylaxis.[3] This, with the ionising power alluded to above, explains why light proves of service in a number of widely-differing dermatoses.

It has been used continuously since its introduction by Finsen in 1897 for lupus, and it still remains the best treatment for this complaint, where time and money are of no object.

Much more stress is now laid, however, on the general treatment of the whole body by light than merely the local application to the diseased areas themselves. And in practice it is assisted by local caustic methods, such as the pyotropin mentioned above. All forms of tuberculosis of the skin are now being treated by this general light bath method, as of course are other tuberculous affections.

To mention one other disease which is often amenable to light treatment, I would cite alopecia. Here cases of widely differing causation will sometimes respond to the nutritional stimulus of the light.

But I have tired you too much already for me to enumerate all the conditions which light may benefit. Nor have I time to allude to $CO_2$ snow treatment of nævi, the thallium treatment of ringworm, etc. But I hope that this brief summary of a few of the advances in dermatology may have interested you, and shown at least that the subject has not been allowed to remain stagnant, but that in it as large strides have been made as in other branches of medicine.

REFERENCES.

[1] *Anaphylaxis and Sensitisation.*

[2] An alkaline phenol compound (see Beatty, *Brit. M. J.*, Jan. 14th, 1928).

[3] Bryce, *Brit. M. J.*, March 19th, 1927.

